# Sharp turns and gyrotaxis modulate surface accumulation of microorganisms

**DOI:** 10.1073/pnas.2206738119

**Published:** 2022-10-11

**Authors:** Li Zeng, Weiquan Jiang, Timothy J. Pedley

**Affiliations:** ^a^State Key Laboratory of Simulation and Regulation of Water Cycle in River Basin, China Institute of Water Resources and Hydropower Research, Beijing 100038, China;; ^b^Department of Hydraulic Engineering, Tsinghua University, Beijing 100084, China;; ^c^Department of Applied Mathematics and Theoretical Physics, University of Cambridge, Cambridge CB3 0WA, United Kingdom

**Keywords:** swimming microorganisms, surface accumulation, gyrotaxis, rotational diffusion

## Abstract

The accumulation of swimming microorganisms at surfaces is an essential feature of various physical, chemical, and biological processes in confined spaces. To date, this accumulation is mainly assumed to depend on the change of swimming speed and angular velocity caused by cell-wall contact and hydrodynamic interaction. Here, we measured the swimming trajectories of *Heterosigma akashiwo* (a biflagellate marine alga) near vertical and horizontal rigid boundaries. We observed that the probability of sharp turns is greatly increased near a vertical wall, resulting in significant changes in the distributions of average swimming speed, angular velocity, and rotational diffusivity near the wall (a quantity that has not previously been investigated) as functions of both distance from the wall and swimming orientation. These cannot be satisfactorily explained by standard hydrodynamic models. Detailed examination of an individual cell trajectory shows that wall contact by the leading flagellum triggers complex changes in the behavior of both flagella that cannot be incorporated in a mechanistic model. Our individual-based model for predicting cell concentration using the measured distributions of swimming speed, angular velocity, and rotational diffusivity agrees well with the experiment. The experiments and model are repeated for a cell suspension in a vertical plane, bounded above by a horizontal wall. The cell accumulation beneath the wall, expected from gyrotaxis, is considerably amplified by cell-wall interaction. These findings may shed light on the prediction and control of cell distribution mediated by gyrotaxis and cell-wall contact.

## Significance

Populations of swimming microorganisms are ubiquitous in aqueous environments from blood vessels to oceans and from biofilms to biotechnological industries, where they routinely encounter solid boundaries. This paper explores experimentally how the presence of boundaries influences the behavior of a marine alga (*Heterosigma akashiwo*), whose normal trajectories exhibit both random sharp turns and gravitational reorientation (gyrotaxis). Proximity to a plane boundary strongly increases the probability of sharp turns and thereby influences the distributions of swimming speed, angular velocity and, unexpectedly, rotational diffusivity as functions of distance from the boundary and of swimming orientation. These variations all contribute to enhancing accumulation beneath an upper boundary much more than gyrotaxis alone.

Solid surfaces are ubiquitous features of the environments in which motile microorganisms live, grow, and reproduce (1–4). Whether or not they adhere to the surface, the accumulation of motile cells on or near a surface is a long-recognized phenomenon for many biological processes occurring at the surface, such as the formation of bacterial biofilms (5–7); attached algal cultivation for biofuels (8); sperm transport within the female reproductive tract (9–11); and harmful algal blooms in rivers, lakes, and oceans (12, 13). Understanding the mechanisms driving such accumulation is significant for predicting its biological implications.

Earlier work on the accumulation of swimming microorganisms near a surface can be traced back to the observation by Rothschild (14) in the 1960s that bull sperm accumulate near a slide and coverslip. Similar phenomena have been found for other motile microorganisms, including bacteria and algae (15–19). Here, we shall concentrate on motile algae, such as *Heterosigma akashiwo* and *Chlamydomonas reinhardtii*, both of which are normally puller-type swimmers unlike bacteria and spermatozoa, most of which are pushers. Three main mechanisms have been proposed for wall accumulation. The first is pure hydrodynamics, in which the proximity of a no-slip wall alters the flow field generated by a swimmer, resulting in a torque that may change the swimmer’s orientation; this mechanism attributes the surface accumulation to long-range hydrodynamic interaction, which tends to reorient tilted pushers to swim parallel to the wall and to redirect pullers to approach or leave the wall perpendicularly (15). The second is response to an external field, such as gravity, in which cells swim up (negative gravitaxis) or down (positive gravitaxis) in still water and accumulate at nonvertical boundaries. The reason for cells to swim up (or down) may be shape asymmetry (16) or density inhomogeneity (e.g., bottom heaviness) (20); when a cell is displaced from a vertical orientation, it experiences a gravitational torque that tends to restore it to the vertical at a rate governed by the hydrodynamic resistance to rotation. The orientational response to the gravity-viscous torque balance is known as gyrotaxis (21). The third is contact with the wall by the cell body or its propulsive organelles (cilia or flagella) (22, 23). In this case, accumulation is attributed to the preferential surface scattering (24, 25) and sliding (18, 26) caused by collisions with the surface (17).

In ref. 23, measurements were reported of the three-dimensional trajectories of microswimmers near the top and bottom rigid walls of a chamber containing a dilute suspension of *C. reinhardtii*. The authors examined many trajectories within 30 µm of the wall and found that cells that approached the wall at shallow angles (less than about $20^{{}^{\circ}}$) tended to reflect smoothly from the wall at approximately the same angle and speed, exhibiting a relatively weak wall repulsion, consistent with the hydrodynamic theory of ref. 27. On the other hand, cells that approached more steeply would first be attracted to the wall and then, very near the wall, reflect at an angle (approximately $20^{{}^{\circ}}$) that was independent of the angle of incidence; these cells’ trajectories showed evidence of wall contact, such as a rapid deceleration followed by acceleration and a sharp discontinuity in angular velocity. The duration of the contact or residence time ($\tau_{\text{res}}$) was generally of the order of 1 s. These authors also measured the cell concentration as a function of the wall-normal distance (*z*), over a much larger range of *z*, and found significant accumulation of cells within that range. The measured distribution could not be explained mechanistically using standard hydrodynamic theory (15) nor by accounting for the effective diffusion caused by cells’ random swimming. The authors were able to recover the distribution by using a Markov Chain Monte Carlo simulation, in which the cell swimming directions were randomly reoriented at each time step by an angle of which the probability density function (PDF) was taken from their measurements. However, they did not explain the physical mechanism leading to this wall-attractive reorientation. Moreover, they did not mention any effect of gravity, such as gravitaxis or gyrotaxis, as a possible cause of wall accumulation (21).

Here, we perform an experimental examination of the interaction and accumulation at plane walls of a different gyrotactic motile alga, *H. akashiwo*. This is a marine species, often responsible for harmful algal blooms (12). *H. akashiwo* has two flagella, only one of which is used for propulsion, normally as a puller, and the other of which is used to influence the cell’s orientation. We found that the probability of sharp turns and the swimming direction after a turn are significantly influenced by the presence of a wall, resulting in intense changes in the distributions of average swimming speed, angular velocity, and rotational diffusivity. Detailed examination of an individual cell trajectory shows that wall contact by the leading flagellum triggers complex responses in the behavior of both flagella. The excellent agreement between an individual-based model (28, 29) and our experiments reveals that the strong inhomogeneity of rotational diffusivity near the wall strongly affects cells’ distribution beneath a horizontal wall and that the surface accumulation expected from gyrotaxis beneath a horizontal wall can be considerably amplified by cell-wall interaction.

## Results

### Swimming Trajectories near a Vertical Wall.

**Fig. 1.**
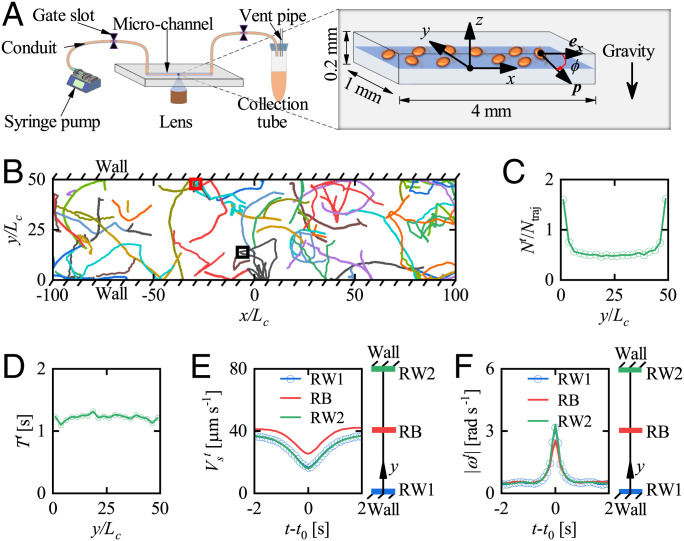
Observation of cells’ trajectories in the horizontal plane (*x*–*y*). (*A*) Schematic of the experimental setup for observing cell swimming in the horizontal plane and the perspective view of imaging area with length 4 mm, width 1 mm, and height 200 µm. Cells’ trajectories are recorded at 10 frames per second using a 4× lens. The angle ϕ defines the cell orientation, p, in the horizontal plane. The direction of gravity is along the negative *z* direction. (*B*) Samples of trajectories. The trajectories enclosed by the black and red squares represent sharp turns in the bulk and near the wall, respectively. *L_c_* (20 µm) is the characteristic length of *H. akashiwo*, approximately equal to half the cell body size plus the length of the one flagellum that provides the main pulling force. (*C*) The variation of $N_{t}/N_{\text{traj}}$ with locations across the channel, $y/L_{c}$, where *N^t^* and $N_{\text{traj}}$ represent the numbers of sharp turn events and trajectories in each space interval of $2.5L_{c}$, respectively. (*D*) Variation of the mean duration of sharp turns, *T^t^*, with, $y/L_{c}$. *E* and *F* show the variation of region-averaged swimming speed, $V_{s}^{t}$, and angular velocity magnitude, $\left|\omega^{t}\right|$, with time, $t-t_{0}$, during a sharp turn for different regions, where *t*_0_ represents the moment at which cells’ angular velocity is maximum during the sharp turn. RW1 and RW2 represent regions next to the walls: $0\leq y/L_{c}\leq 2.5$ and $47.5\leq y/L_{c}\leq 50$, respectively. RB represents the region in the bulk almost unaffected by the wall: $23.75\leq y/L_{c}\leq 26.25$.

In order to explore cells’ swimming behavior near a vertical wall, we employed user-customized micrometer-resolution particle image velocimetry (micro-PIV) to observe the motility of *H. akashiwo* in a horizontal slice in the center of a rectangular channel (Fig. 1*A* and *Materials and Methods*). The characteristic length *L_c_* (20 µm) of a cell is approximately equal to half the cell body length plus the length of one flagellum. Regarding the orientation, ϕ, of *H. akashiwo* in the horizontal plane, we chose 0<ϕ<π for cells that are swimming toward the wall at *y* = 0 and away from the “far” wall at $y/L_{c}=50$ and −π<ϕ<0 for cells swimming away from *y* = 0 and approaching the far wall. In general, the cells have three-dimensional trajectories and therefore swim through the imaging slice (the optical depth of field was 30.8 µm—±15.4 µm on either side of the object plane in focus); we record and analyze only those trajectories that remain in the slice throughout. A number of these two-dimensional trajectories are shown in Fig. 1*B*. It can be seen that many trajectories far from the wall remain straight or gently curved, while others appear to zigzag because they are in fact helical. Both types have occasional sharp changes of direction (*SI Appendix*, Fig. S1); near the wall, such changes always occur unless the incoming angle with the wall is very small.

An example trajectory is marked by the small red rectangle in Fig. 1*B*. Far from the wall, this trajectory is helical with a straight helical axis, but after a sharp turn, the cell swims parallel to the wall for some time before moving away after another sharp turn (*SI Appendix*, Fig. S1*B*). Such swimming behavior, parallel to the wall after touching the surface, has also been reported in refs. 18, 24, 25, and 30. Here, a sharp turn is deemed to occur when the magnitude of the angular velocity exceeds a critical value $\omega_{c}^{t}=\pi /8\;\text{rad}\;{\text{s}}^{\text{-}1}$, which is much greater than the mean value ($0.04\;\text{rad}\;{\text{s}}^{\text{-}1}$) in the bulk. The sensitivity of the number of recorded sharp turns to $\omega_{c}^{t}$ is illustrated in *SI Appendix*, Fig. S2. [Sharp turns have also been observed for *C. reinhardtii* (31).] It is found that the probability of sharp turns occurring near the wall is greater than that far from the wall (Fig. 1*C*). The enhanced local likelihood of sharp turns results in more sharp turn events near the wall (*SI Appendix*, Fig. S3*A*), although the number of observed trajectories near the wall is less than in the bulk (*SI Appendix*, Fig. S3*B*). However, the mean duration of each sharp turn, *T^t^*, is independent of the distance of a cell from the wall (Fig. 1*D*). The magnitudes of the average angular velocity during each sharp turn, *ω^t^*, are almost the same for all sharp turns (Fig. 1*F*), while the average swimming speed during each sharp turn, $V_{s}^{t}$, remains qualitatively the same for all sharp turns (and is only slightly higher in the bulk than in the wall regions) (Fig. 1*E*). Within a distance of *L_c_* from the wall, these events signal contact of the locomotory flagellum with the wall, after which the cell reorients itself and, when oriented away from the wall, it gradually speeds up again. The cause of sharp direction changes in trajectories far from the wall (e.g., the one marked with a black rectangle in Fig. 1*B*) is less clear; we hypothesize that these are caused by contact of the swimming flagellum with foreign bodies, either other cells or inert particles in the suspension.

**Fig. 2.**
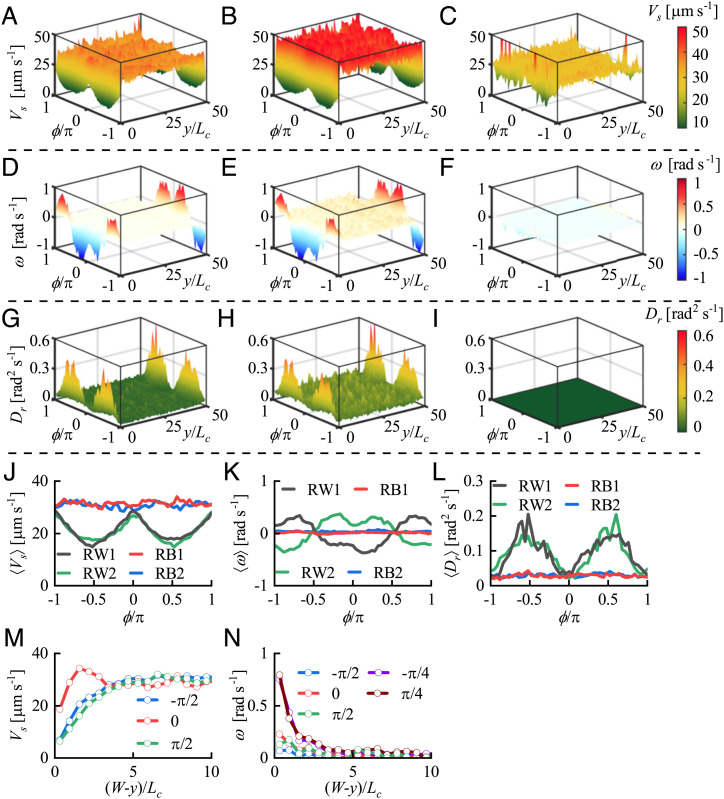
The effects of sharp turns on the ensemble-averaged swimming speed *V_s_*, angular velocity *ω*, and rotational diffusivity *D_r_*. *A*, *D*, and *G* present the variation of *V_s_*, *ω*, and *D_r_* with lateral location $y/L_{c}$ and orientation ϕ/π, where *L_c_* (20 µm) is the characteristic length of *H. akashiwo*, approximately equal to half the cell body size plus the length of one flagellum. *B*, *E*, and *H* present the same data for those trajectories that contain sharp turns. *C*, *F*, and *I* present the data for the trajectories that do not contain sharp turns. The variation of *V_s_*, *ω*, and *D_r_* near the wall is mainly influenced by sharp turn events. Here, the critical value of angular velocity, $\omega_{c}^{t}=\pi /8\;\text{rad}\;{\text{s}}^{\text{-}1}$, is used to identify the sharp turns. (*J*–*L*) The spatially averaged swimming speed $\langle V_{s}\rangle$, angular velocity 〈ω〉, and rotational diffusivity $\langle D_{r}\rangle$ in regions RW1 (gray line), RB1 (red line), RB2 (blue line), and RW2 (green line). RW1 and RW2 denote the regions next to the wall: $0\leq y/L_{c}\leq 2.5$ and $47.5\leq y/L_{c}\leq 50$, respectively. RB1 and RB2 denote the regions almost unaffected by the wall: $5\leq y/L_{c}\leq 10$ and $40\leq y/L_{c}\leq 45$, respectively. (*M* and *N*) *V_s_* and *ω* near the wall $y/L_{c}=50$ for various swimming directions ϕ: −π/2 (blue line marked by blue circles), −π/4 (purple line marked by purple circles), zero (red line marked by red circles), π/4 (brown line marked by brown circles), and π/2 (green line marked by green circles).

Next, we investigate the effects of the cell-wall interaction on the swimming behavior of the population. To obtain statistics for this behavior, we took the measured cell trajectories, smoothed them, and converted them to the following ensemble-averaged quantities as functions of their orientation ϕ as well as the distance from the wall *y*: swimming speed *V_s_*, angular velocity *ω*, and rotational diffusivity *D_r_* (*Data Analysis of Experimental Results*, Fig. 2 *A*, *D*, and *G*, and *SI Appendix*, Fig. S4). The experimental results show that these quantities remain uniform in the bulk (*ω*  =  0, for example) but vary dramatically in the proximity of the wall. The frequency of sharp turns is almost uniform in the bulk (Fig. 1*C*), where cells can turn in an arbitrary, presumably isotropic direction. The increase in the number of sharp turns near the wall is obvious from *SI Appendix*, Fig. S3*A*, and it is observed that some cells prefer to swim parallel to the wall after they collide with it (*SI Appendix*, Fig. S1*B*). The question arises as to whether the changed frequency of sharp turns and the variation of cell behavior during sharp turns are caused by the cell-wall interaction. If so, how does this determine the average swimming behavior of *H. akashiwo*? To address this issue, we examined the contribution of sharp turns to *V_s_*, *ω*, and *D_r_*. The plots in Fig. 2 *A*, *D*, and *G* are the data on the ensemble-averaged swimming speed, angular velocity, and rotational diffusivity, for all the trajectories, plotted as functions of *y* and ϕ. The plots in Fig. 2 *B*, *E*, and *H* show the same quantities for all trajectories containing a sharp turn (defined by the maximum angular velocity exceeding the critical value). The plots in Fig. 2 *C*, *F*, and *I* show the data for all the remaining trajectories. It is clear that the ensemble-averaged swimming behavior of cells is attributable to the occurrence of sharp turns near the wall.

We now investigate whether the variation of ensemble-averaged swimming behavior near the wall can be explained by hydrodynamic theory. The range of cell-wall interaction for swimming speed is dependent on cells’ swimming orientation, ϕ. As cells perpendicularly approach the wall (ϕ=π/2 for the wall at *y* = 0 and ϕ=−π/2 for the wall at $y/L_{c}=50$), their swimming speed in the horizontal plane, *V_s_*, decreases as the distance from the wall, *h*, decreases below approximately 100 µm. As cells swim parallel to the wall, the influenced distance is approximately 70 µm from the wall (Fig. 2*M* and *SI Appendix*, Fig. S4*A*). The variation with *h* of $-(V_{s}-V_{sb})\sin\;\phi$ (corresponding to the wall-induced velocity in ref. 15), where *V_sb_* is the bulk swimming speed, does not exhibit the characteristic $h^{-2}$ dependence and changes between attraction and repulsion at the orientations $\phi =\pm \pi /2\pm \arccos\;(1/\sqrt{3})$, which are representative of the far-field hydrodynamic effect of an image force dipole (*SI Appendix*, Fig. S5 *A* and *C*) (15).

The mean angular velocity is zero in the bulk but is clearly nonzero near the walls (Fig. 2*K* and *SI Appendix*, Fig. S4*B*), which means that cells near the wall effectively experience a wall-induced external torque. It can be seen that the angular velocity of a cell depends not only on its distance from the wall, but also on its swimming direction ϕ (Fig. 2 *K* and *N*). The maximum angular velocity (of the order of $1\;\text{rad}\;{\text{s}}^{\text{-}1}$) occurs at a tilted direction (approximately ϕ=±π/4 or ϕ=±3π/4) rather than a direction parallel or perpendicular to the wall. If the wall effect was caused by a tilted image force dipole, the wall-induced angular velocity, ω(y,ϕ), for a puller-type microorganism could be expressed in the present notation (15) as

[1] $$\omega (h,\phi )=\frac{p}{8\pi \mu }\frac{3\sin\;(2\phi )}{16h^{3}}\left[1+\frac{\Gamma }{2}\left(1+{\sin\;}^{2}\phi \right)\right],$$

where p (<0) is the dipole strength, *μ* is the dynamic viscosity of the fluid, and $\Gamma =(r^{2}-1)/(r^{2}+1)$, where *r* is the aspect ratio of the cell, which is approximately equal to zero for an almost spherical puller, such as *H. akashiwo*. Eq. **1** shows that an outgoing swimmer would tend to leave the wall at a right angle, which is in qualitatively good agreement with the present measurements. However, for an incoming swimmer, it is inconsistent with the experimental result that the cell tends to approach the wall at a small angle ϕ (*SI Appendix*, Fig. S5 *B* and *D*).

We also compared the normalized ensemble-averaged angular velocity of *H. akashiwo* very close to the wall ($y/L_{c}\leq 0.625$) given by lubrication theory for the near field of a spherical (puller) squirmer (32, 33) and experimental observation (*SI Appendix*, Fig. S6). It is shown that the variation of angular velocity with the orientation, ϕ, still cannot be well described by the lubrication results, in particular for 1) the angle corresponding to the maximum angular velocity, 2) cells or squirmers swimming away from the wall (ϕ<0), and 3) more vigorous swimming (larger values of the squirming parameter *β*). Moreover, the thickness of the region of strong angular velocity variation (≈50 µm) is greater than *L_c_*. Hence, the effective torque experienced by a cell at a distance greater than *L_c_* is neither purely hydrodynamic nor a direct consequence of wall contact.

**Fig. 3.**
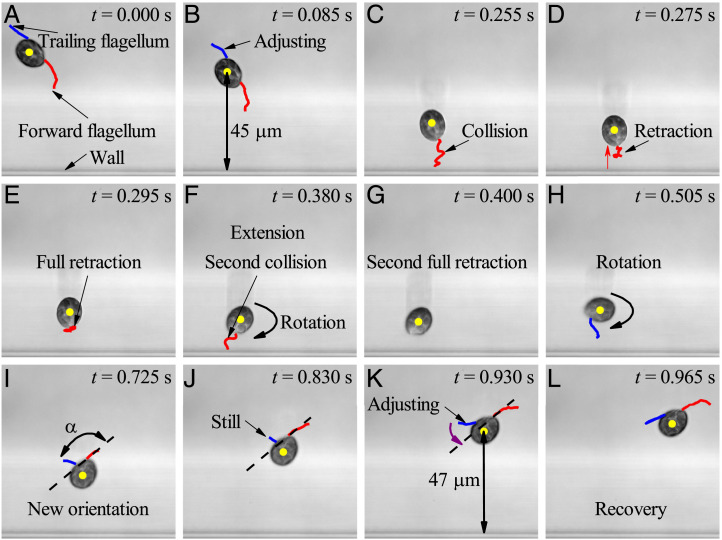
Process of a sharp turn of *H. akashiwo* in the region close to a wall taken from Movie S1. The wall greatly affects the motility of both forward and trailing flagella. (*A*) The normal swimming mode of *H. akashiwo* almost unaffected by the wall. The red and blue curves represent the forward and trailing flagella, respectively. The yellow point denotes the centroid of the cell. (*B*) The swimming pattern of the cell at a distance larger than the characteristic length ($L_{c}=20$ µm). The swing amplitude and frequency of the trailing flagellum change before the forward flagellum touches the wall. (*C*) The first collision of the forward flagellum with the wall. The trailing flagellum is not in the focal plane. (*D*) The retraction of the forward flagellum. After the forward flagellum touches the wall, it retracts quickly to avoid further collision of the cell body with the wall. (*E*) The first full retraction of the forward flagellum. During the retraction, the orientation of the cell does not change much. (*F*) The second collision of the forward flagellum with the wall. After fully retracting the forward flagellum from the wall, the cell begins to rotate and extend its forward flagellum to pull itself. (*G*) The second full retraction of the forward flagellum. After the forward flagellum hits the wall the second time, it retracts from the wall again. (*H*) The rotation of the cell after the second full retraction. The trailing flagellum is not parallel to the long axis of the cell. (*I*) The stable orientation following the second contact with the wall. The cell leaves the wall at this angle. The angle between the forward and trailing flagella, *α*, is slightly larger than π/2 rather than *π* as it is in free space. (*J*) After the cell achieves a stable orientation, *α* does not change significantly. (*K*) The change of the angle between the forward and trailing flagella. *α* gradually increases toward *π*. The maximum distance from the wall of the cell’s centroid during this stage is about 50 µm, 2.5 times the characteristic length *L_c_*. (*L*) The recovery of cell swimming mode. The swimming mode of both the forward and trailing flagella seems unaffected by the wall, with *α* tending to *π*.

We hypothesize that the flagella beat pattern must be changed near the wall. To examine this hypothesis, we have observed the swimming behavior of a small number of cells using an ultrahigh-resolution microscope (DeltaVision OMX V4; GE Healthcare; 60× magnification and 200 frames per second) (*Materials and Methods*). The details for just one trajectory are given in Fig. 3. First, we found that in the region with $y/L_{c}\leq 1$, the forward flagellum retracts quickly and reorients itself to avoid the further collision of the cell body with the wall after it touches the wall (Fig. 3 *C*–*F*). During this stage, the cell’s swimming speed decreases abruptly to its minimum value and increases gradually again. In such a case, the swimming speed and angular velocity cannot be well described by near-field theory, such as lubrication theory. Second, we observed two consecutive collisions of the forward flagellum with the wall, which means that the reorientation caused by sharp turns due to the cell-wall interaction is limited by the presence of the wall (Fig. 3 *C*–*I*). This is the reason that *H. akashiwo* tends to swim parallel to the wall or at a small angle with the wall. Third, we observed that the swaying behavior of the trailing flagellum of the cell changes before and after a collision with a wall even if its distance from the wall is greater than *L_c_* (Movie S1). When *H. akashiwo* approaches the wall with distance larger than *L_c_*, its amplitude and beating frequency change. When it leaves the wall, the angle between the forward and trailing flagella (approximately π/2) does not recover until its distance from the wall is larger than $2.4L_{c}$. This means that cells’ swimming behaviors, due to the presence of the wall, change even if the forward flagellum does not contact the wall. The traditional near- and far-field theories are unable to predict the ensemble-averaged swimming speed and angular velocity because neither of them can take the changes of flagella beating into account.

Typically, the effective rotational diffusivity has been assumed to be constant irrespective of the distance from a wall. However, our experiments showed that cells’ rotational diffusion is greatly enhanced by the presence of the wall, and the maximum rotational diffusivity near the wall is larger by an order of magnitude than that ($\approx 0.025\;{\text{rad}}^{2}\;{\text{s}}^{-1}$) in the bulk. Also, the cells with orientation perpendicular to the wall show stronger randomness than those parallel to the wall (Fig. 2*L* and *SI Appendix*, Fig. S4*C*). Such variation of rotational diffusivity with space and orientation is found to play a significant role in determining the cells’ concentration distribution, which will be described in the subsequent section.

### Cell Concentration Distribution near a Vertical Wall.

**Fig. 4.**
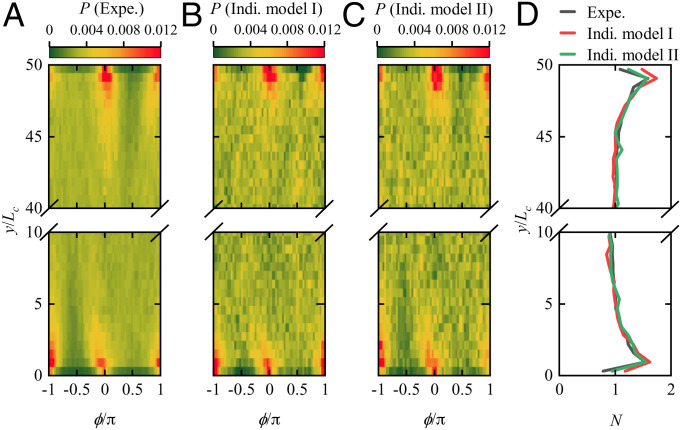
Distribution of *H. akashiwo* in the horizontal plane. (*A*) The experimentally (Expe.) measured PDF to find a cell at the horizontal position, $y/L_{c}$, and the swimming direction, ϕ/π. (*B*) The computed PDF based on the individual (Indi.) model I (constant rotational diffusivity). (*C*) The computed PDF based on the individual model (space-varying rotational diffusivity). (*D*) Comparison of computed and measured normalized concentrations, *N*. The gray line represents the experimental result, while the red and green lines denote the numerical results given by the individual models I and II, respectively.

To explore the effect of the changes of swimming behavior caused by cell-wall interaction on cells’ distribution, we investigated the PDF P(y,ϕ) for finding a cell in position *y* at orientation ϕ and the orientation-averaged and normalized cell concentration $N(y)=N^{*}(y)/{\bar{N}}^{*}\;(N^{*}(y)=\int_{-\pi }^{\pi }P(y,\phi )\;\text{d}\phi$, and the overbar represents the width average of $N^{*}(y))$ at position *y* based on the statistical analysis of measured trajectories (this is the concentration measure that would be predicted by a continuum model). Cell accumulation is found to appear in the vicinity of the wall, the distance from the wall (*δ*) of peak concentration being about 20 µm ($y/L_{c}\approx 1$) (Fig. 4 *A* and *D*). The accumulation of *H. akashiwo* (different strain) near a vertical wall has also been reported in ref. 18. Similar phenomena were found for the curvature-guided distribution of *C. reinhardtii* in a circular compartment with δ≈10 µm (30), approximately the cell body size. Also, the cell concentration displays nonuniformity in the orientation space. The dominant swimming direction of *H. akashiwo* near the wall is parallel to the wall (ϕ=0 and *π*), in contrast to the unbiased swimming direction in the bulk. Furthermore, some slight differences can be found between the concentration distributions around ϕ=π/2 and −π/2, which implies that cells have a relatively larger chance of approaching the wall perpendicularly than of departing from the wall perpendicularly.

What is responsible for the concentration distribution in the region adjacent to the wall? To address this question, we used an individual-based model to examine the effects of the cell-wall interaction on cells’ distribution. For cell motion in the horizontal plane, the individual (Indi.) model is given by

[2] $$\{\begin{array}{l} \frac{\text{d}y}{\text{d}t}=-V_{s}(y,\phi )\sin\;\phi ,\\ \text{d}\phi =\omega (y,\phi )\text{d}t+\sqrt{2D_{r}(y,\phi )}\text{d}W, \end{array}$$

where *W* is white noise (to be interpreted in the Itô sense). In the individual model, if we ignore the variation of swimming speed, angular velocity, and rotational diffusivity with distance from the wall due to cell-wall interaction (i.e., $V_{s}=V_{sb}$, *ω*  =  0, and $D_{r}=D_{rb}$, where *D_rb_* is the rotational diffusivity in the bulk), the PDF is uniform for all positions and directions, which is qualitatively inconsistent with our experimental observation (Fig. 4 *A* and *D*). After we include the wall-induced variation of swimming speed and angular velocity given by direct experimental observation (*SI Appendix*, Fig. S4 *A* and *B*), the simulated PDF (Fig. 4 *B* and *D*) is qualitatively in good agreement with the experimental observation (Fig. 4 *A* and *D*). However, there still exist some differences between computed and measured P(y,ϕ) and *N*(*y*) in the region where the peak concentration occurs. After we include the variation of rotational diffusivity near the wall, the simulated PDF (Fig. 4 *C* and *D*) is in better agreement with the experimental observation (Fig. 4 *A* and *D*). Such effects of rotational diffusion on cells’ distribution become more significant in the vertical plane, which will be shown in detail subsequently. Thus, the cells’ horizontal distribution near a vertical wall is essentially determined by the effect of the cell-wall interaction on the swimming speed, angular velocity, and rotational diffusivity and hence, on the change in the number of and reorientation caused by sharp turns due to cell-wall interaction.

### Swimming Behavior near a Horizontal Wall.

**Fig. 5.**
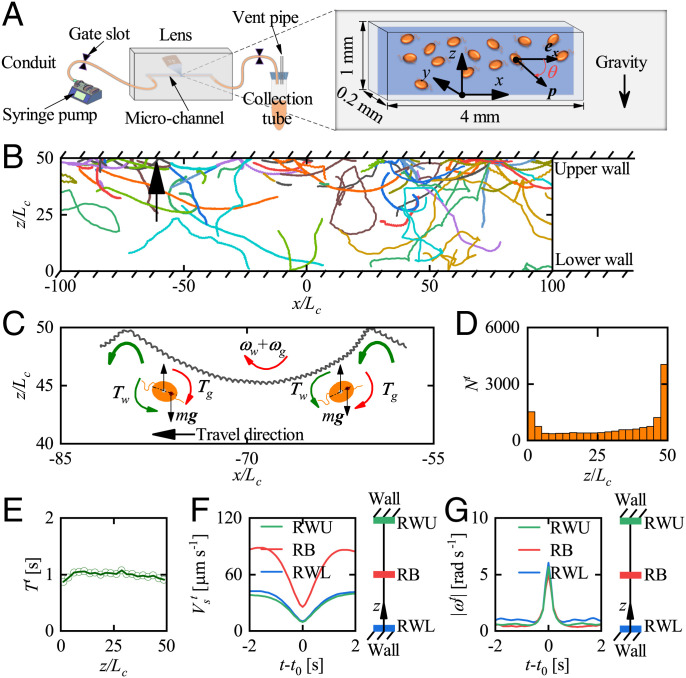
Observation of cells’ trajectories in the vertical plane (*x*–*z*). (*A*) Schematic of the experimental setup for observing cell swimming in the vertical plane and a perspective view of the imaging area. The microchannel and imaging area are the same as those for the horizontal observation, but they are rotated 90${}^{{}^{\circ}}$ around the *x* axis to examine swimming behaviors of *H. akashiwo* in the vertical plane. The angle *θ* defines the cell orientation, p, in the vertical plane. (*B*) Samples of trajectories. Many approximately cycloidal trajectories can be found beneath the upper wall: for example, the gray one indicated by the black arrow. (*C*) Expanded view of the gray trajectory in *B*. The curvature is attributed to the angular velocity *ω_w_* caused by the torque *T_w_* and the angular velocity *ω_g_* caused by the gravitational torque *T_g_*. The attenuation of swimming speed near a horizontal wall is similar to that near the vertical wall. (*D*) The number of sharp turn events, *N_t_*, occurring at different vertical locations across the channel. The critical value of angular velocity, $\omega_{c}^{t}=\pi /2\;\text{rad}\;{\text{s}}^{\text{-}1}$, is used to identify the sharp turns, which is well above the typical value ($0.1\;\text{rad}\;{\text{s}}^{\text{-}1}$) of angular velocity due to gyrotaxis. (*E*) The mean duration of a sharp turn, *T^t^*, at different locations. The variation of (*F*) the region-averaged swimming speed, $V_{s}^{t}$, and (*G*) the region-averaged magnitude of angular velocity, $\left|\omega^{t}\right|$, with time, $t-t_{0}$, during a sharp turn for different regions, where *t*_0_ represents the moment at which cells’ angular velocity is maximum during the sharp turn. RWL and RWU represent the regions immediately connected to the wall: $0\leq z/L_{c}\leq 2.5$ and $47.5\leq z/L_{c}\leq 50$, respectively. RB represents the region in the bulk almost unaffected by the wall: $23.75\leq z/L_{c}\leq 26.25$.

We now consider how gyrotaxis affects the swimming behavior of *H. akashiwo*. To explore this issue, we repeat the previous experiments in a vertical plane with horizontal boundaries using the same chamber rotated by π/2 radians (Fig. 5*A*). We denote by *θ* the orientation angle in the vertical plane measured clockwise below the horizontal (i.e., away from the top wall), which means that we chose 0<θ<π for cells that are swimming toward the wall at *z* = 0 and away from the far wall at $z/L_{c}=50$ and −π<θ<0 for cells swimming away from *z* = 0 and approaching the far wall. A number of two-dimensional trajectories are shown in Fig. 5*B*. It can be seen that the trajectories are concentrated near the upper wall ($z/L_{c}=50$) rather than the lower wall ($z/L_{c}=0$). From now on, we mainly consider the upper wall. Many trajectories near the upper wall appear approximately cycloidal, with long stretches of gentle, concave-upward curvature separated by relatively sharp, concave-downward turns when the cells approach the wall (Fig. 5*B*).

This phenomenon can be explained by a combination of sharp turns, similar to those observed in the horizontal plane near a vertical wall and discussed in the previous subsections, and gyrotaxis. To provide an intuitive picture, one typical cell trajectory observed in the experiment is depicted in Fig. 5*C*. Cells swimming nonvertically far from the wall tend to swim upward due to the gravitational torque *T_g_* (negative gravitaxis), resulting in a concave-up trajectory. However, in the region contiguous with the upper wall, the trajectory changes from concave up to concave down. The reason must be that the cell experiences a strong torque *T_w_* opposite to *T_g_*, which rotates the cell to swim downward. When the cell has swum down below the wall region, it will rotate toward up swimming again as *T_w_* is weaker than *T_g_*. As for the sharp turns in horizontal trajectories, the number of sharp turns in the vertical plane is greater in the regions very near the horizontal walls than just outside those regions. However, the number of sharp turns does not remain constant across the bulk of the chamber but increases (gradually) with increasing $z/L_{c}$ (Fig. 5*D*); this is a consequence of the increase in cell concentration with height (see below). The probability of sharp turns in the bulk still remains uniform (*SI Appendix*, Fig. S7). Here, the critical value of angular velocity, $\omega_{c}^{t}=\pi /2\;\text{rad}\;{\text{s}}^{\text{-}1}$, is used to identify the sharp turns, which is well above the typical value ($0.1\;\text{rad}\;{\text{s}}^{\text{-}1}$) of angular velocity due to gyrotaxis. Note that the region next to the bottom wall contains a much smaller number of cells than the region next to the top wall and therefore is the site of many fewer sharp turns. However, it is notable that the mean duration of a sharp turn *T^t^* (Fig. 5*E*), the swimming speed during a sharp turn $V_{s}^{t}$ (Fig. 5*F*), and the magnitude of the angular velocity during each sharp turn $\left|\omega^{t}\right|$ (Fig. 5*G*) remain qualitatively the same (Fig. 1 *D*–*F*). This suggests that gyrotaxis mainly changes the location of cells’ sharp-turn events.

**Fig. 6.**
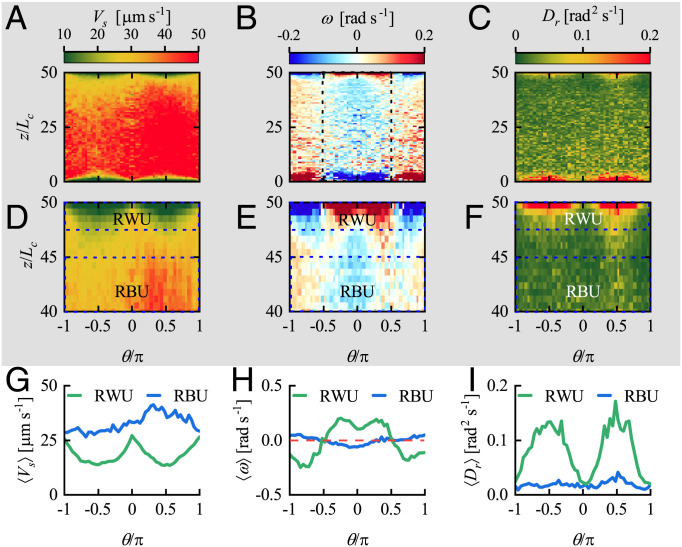
Swimming behavior of *H. akashiwo* in the vertical plane. The variation of the statistically averaged (*A*) swimming speed *V_s_*, (*B*) angular velocity *ω*, and (*C*) rotational diffusivity *D_r_* with the vertical position $z/L_{c}$ and swimming direction θ/π. RWU and RBU denote the same regions as RW2 and RB2 in *SI Appendix*, Fig. S4 but in the vertical plane. *D*–*F* are the expanded views of the region near the upper wall for *A–C*, respectively. The spatially averaged (*G*) swimming speed $\langle V_{s}\rangle$, (*H*) angular velocity 〈ω〉, and (*I*) rotational diffusivity $\langle D_{r}\rangle$ in regions RBU (blue line) and RWU (green line).

To elucidate the overall effect of gyrotaxis on the cell’s swimming behavior, we plotted the ensemble-averaged swimming speed $V_{s}(z,\theta )$, angular velocity ω(z,θ), and rotational diffusivity $D_{r}(z,\theta )$ (Fig. 6). The strong variation of swimming speed, angular velocity, and rotational diffusivity occurs near the top and bottom walls for vertical swimming in the vertical plane as they do near the side walls for horizontal swimming. The thickness of the zone in which these physical quantities vary strongly, for swimming in the vertical plane, is approximately 50 µm. This is comparable with that for swimming in the horizontal plane, which implies that the cell-wall interaction is dominant over gyrotaxis near the walls. However, the cells’ swimming speed is found to be weakly anisotropic far from the walls, the mean swimming speed upward being less than that downward (Fig. 6 *A*, *D*, and *G* compared with Fig. 2*A* and *SI Appendix*, Fig. S4*A*). Although the cells’ swimming speed in the bulk in the vertical plane is greater than that in the horizontal plane, the swimming speed is comparable for all cells in the whole width and height (*SI Appendix*, Fig. S8). The reason is that the number of cells in the bulk is much less than that near the wall for the vertical plane because only cells with strong swimming speed can reach the bulk due to gyrotaxis. Unlike the angular velocity for the swimming in a horizontal plane, which is isotropic in the bulk, the angular velocity varies with *θ* in the vertical plane, being negative for −π/2≤θ≤π/2 and positive for −π≤θ<−π/2 and π/2<θ≤π (Fig. 6 *B*, *E*, and *H*), since gyrotaxis of *H. akashiwo* tends to orient the cells toward upward swimming. The angular velocity in the bulk can be well fitted using the formula of angular velocity due to gravity [ω=−cos θ/(2B), where *B* is the timescale for cell reorientation by the gravitational torque against the viscous resistance] (*SI Appendix*, Fig. S9). Cells’ gyrotaxis can weaken and enhance the angular velocity caused by cell-wall interaction to a certain extent at the top and bottom walls, respectively, although such effects cannot qualitatively change cells’ rotation due to cell-wall interaction. The distribution of rotational diffusivity in the vertical plane (Fig. 6 *C*, *F*, and *I*) is similar to that in the horizontal plane (Fig. 2 *G* and *L*), with the maximum occurring when cells approach the wall.

### Cell Concentration Distribution near Horizontal Walls.

**Fig. 7.**
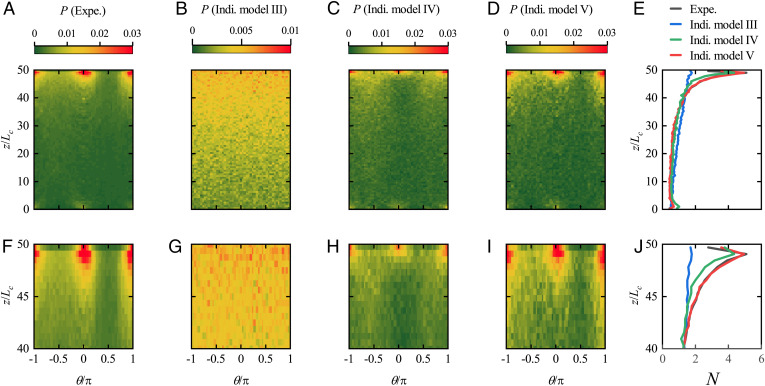
Distribution of *H. akashiwo* in the vertical plane. (*A*) The experimentally (Expe.) measured PDF, $P(z/L_{c},\theta /\pi )$, to find a cell at the vertical position, $z/L_{c}$, and the swimming direction, θ/π. Cells tend to accumulate beneath the upper wall with a dominant swimming direction parallel to the wall. (*B*) The computed *P* based on the individual (Indi.) model (Indi. model III) accounting for gyrotaxis but not cell-wall interaction. Gyrotaxis alone cannot explain the preference of cell swimming direction parallel to the wall in the region immediately connected to the wall. (*C*) The computed *P* based on the individual model (Indi. model IV) accounting for the effects of the cell-wall interaction (neglecting the effect for rotational diffusivity) and the gyrotaxis. (*D*) The computed *P* based on the full individual model (Indi. model V) accounting for gyrotaxis and cell-wall interaction. This model well reproduces the measured *P* in both position and orientation. (*E*) The comparison of computed and measured normalized concentration, $N(z/L_{c})$. The gray, blue, green, and red lines represent $N(z/L_{c})$ generated by the experimental observation and Indi. models III to V, respectively. Indi. models III and IV adopt the same rotational diffusion coefficient, $D_{r}=0.025\;{\text{rad}}^{2}\;{\text{s}}^{-1}$. *F–J* are the expanded views of the region near the upper wall for *A–E*, respectively.

In Fig. 7, we display the measurements and model results for horizontal walls that correspond to Fig. 4 for vertical walls. Fig. 7 *A* and *F* shows the measurements of the PDF P(z,θ) for finding a cell in position *z* at orientation *θ*. It is clear that many more cells accumulate just below the top wall ($z/L_{c}=50$) than above the bottom one ($z/L_{c}=0$) because of their up swimming (negative gravitaxis). A plot of the normalized cell concentration *N*, the normalized integral of *P* over *θ*, is provided in Fig. 7 *E* and *J*. The distance of the concentration peak (*δ*) below the upper wall is about 20 µm, which is approximately *L_c_*, half the cell body size plus the length of a flagellum; this is the same value as found for vertical walls, indicating the same mechanism of cell-wall interaction. Furthermore, the probability density distribution around θ=−π/2 is noticeably greater than that around θ=+π/2. The reason lies in the fact that the cells’ rotation caused by gyrotaxis is in the same sense as the wall-induced rotation around θ=−π/2 but opposite to the wall-induced rotation around θ=+π/2. The variation of concentration with height outside the wall layers follows the expected exponential distribution, $N=N_{0}\exp\;(\sigma z/H)$ for constant *N*_0_ and $\sigma =V_{z}H/D_{T}$, where *D_T_* is the effective translational diffusivity and *V_z_* is the vertical cell velocity in the bulk, both assumed to be constant (16, 21). This formula agrees well with the experimental curve in Fig. 7*E* if *σ*, an effective Péclet number, is equal to 3.112 (*SI Appendix*, Fig. S10).

To investigate the physics behind the observations, we apply the mathematical model with different terms omitted. For the vertical plane, the model consists of Eq. **2** for the horizontal plane, with *y* and ϕ replaced by *z* and *θ*, plus a term in the second equation to take account of gyrotaxis (Eq. **3** in *Mathematical Model*).

The functions we use for swimming speed *V_s_*, angular velocity *ω*, and rotational diffusivity *D_r_* are taken from the measurements in Fig. 6. In this vertical plane, the angular velocity *ω* is equal to the sum of that induced by the wall and that induced by gravity: *ω_w_* and *ω_g_*, respectively. *ω_g_* is computed using [k−(k·p)p]/(2B), where ***p*** is the unit swimming direction and ***k*** is the unit vector directed vertically upward (21).

First, we neglect gravity and consider the contribution of cell-wall interaction alone (Indi. model II). The computed PDF is the same as Fig. 4*C* for both the upper and lower walls, which is clearly inconsistent with the experimental observation that most cells accumulate near the upper wall (Fig. 7*A*); the difference is particularly marked in the plots of *N*(*y*) in Fig. 4*D* and of *N*(*z*) in Fig. 7 *E* and *J*. The cell-wall interaction alone cannot explain the substantial concentration difference across the depth of the microchamber outside the wall regions.

Next, we consider the contribution of gyrotaxis alone by specifying $\omega_{w}=0$ (Indi. model III); the computed PDF and concentration distributions are shown in Fig. 7 *B*, *E*, *G*, and *J*. Although the computed result reflects the increase of cell concentration with *z* across the depth of the chamber outside the wall regions, it is still much less than the observed maximum concentration and cannot reproduce the position and magnitude of the concentration peak.

Now, we consider the combined effects of cell-wall interactions and gyrotaxis by including all the terms *ω_w_*, *ω_g_*, and the variation of *V_s_* (Indi. model IV) but neglecting the effect of cell-wall interaction on rotational diffusion. The computed result is in qualitative agreement with the observation, although the variation of *P* and *N* is obviously less than observed (Fig. 7 *C*, *E*, *H*, and *J*). Finally, we considered the effects of the full cell-wall interaction and gyrotaxis, and again, the results match the experiments (Fig. 7 *D*, *E*, *I*, and *J*). 1) A pronounced peak develops near each wall, 2) the distance of the peaks from the walls is in good agreement with the experiment, and 3) the magnitudes of the peaks are in quantitative agreement. This means that the variation of cells’ rotational diffusion due to cell-wall interaction also plays a significant role in determining cells’ distribution in both position and orientation space. Neglecting the variation of *D_r_* causes an underestimate of the cell concentration beneath a horizontal plane.

Fig. 7 *E* and *J* clearly shows that surface accumulation beneath a horizontal wall, expected from gyrotaxis, can be considerably amplified by cell-wall interaction. The point is that in the bulk, the number of sharp turns is small so that there is only a small, but nonzero, rotational diffusivity. Individual trajectories are, therefore, mostly upward, with zero mean angular velocity. If neither the angular velocity nor the rotational diffusivity are changed near the wall, the only reason for a concentration increase lies in the reflection boundary condition applied at the wall. This is why the concentration distribution for “Indi. model III” in Fig. 7 *E* and *J* shows such a small enhancement just below the wall. The situation is totally changed when the swimming speed, the angular velocity, and the rotational diffusivity are allowed to change near the top wall as observed.

## Discussion

We have experimentally found, as others have (18, 23, 30), that there is accumulation of swimming cells (here, *H. akashiwo*) near plane boundaries, both vertical and horizontal. We have found that the trajectories of swimming cells and the probability of a cell undergoing a sharp turn are significantly influenced by the proximity of a wall. As a result, there is strong variation of ensemble-averaged swimming speed (*V_s_*), angular velocity (*ω*), and rotational diffusivity (*D_r_*) with both the distance from the wall and cell orientation near the wall (the dependence of rotational diffusivity on average local cell orientation is a particular feature of this work). This means that accurate modeling of the cell concentration distribution within a distance of less than $5L_{c}\;(100\;\mu \text{m})$ is not feasible without taking the variation of swimming behavior into account in the governing equations; conventional hard-wall reflection is not applicable. These variations in the inner wall layer (i.e., within a distance of less than *L_c_* from the wall) may be attributed to direct contact of cells or their flagella with the wall. However, the corresponding mechanism is less clear in the outer wall layer (i.e., at distances greater than *L_c_* but less than approximately $5L_{c}$ from the wall) and cannot be well explained, even qualitatively, by conventional hydrodynamics in terms of the force–dipole image system or lubrication theory (15, 32). Near-wall hydrodynamic slip (34) may be one potential mechanism to explain the transition of the behavior of puller-type microorganisms from the bulk to the inner layer, but direct measurement of cell and flagella motion (e.g., Fig. 3) reveals the typical complexity of cells’ behavior as they encounter a wall. Detailed measurement of the flow fields around cells near the wall may be required; experiments to achieve this will shortly be in progress in our laboratory.

The discussion in the above paragraph applies equally to the interaction of cells with vertical or horizontal walls. However, for cells beneath a horizontal wall, the additional factor of gravity (i.e., gyrotaxis) comes into play. The angular velocity *ω* is now the sum of *ω_w_* due to wall interaction, which is zero in the main body of the chamber, and *ω_g_*, which is proportional to the gravitational torque and hence, to −p×g, where **p** is the unit vector in the direction of the cell axis. *ω_g_* is clearly nonzero in the body of the chamber. Thus, outside the wall layers, gyrotaxis operates and leads to the gradual increase of cell concentration *N* with vertical coordinate *z*. Within the wall layers, however, the wall effect dominates the gravitational torque. Gyrotaxis causes cells that leave the wall layer to return to it, which significantly enhances the accumulation of cells that would result from either effect alone.

The details revealed in Fig. 3 show that, after touching the wall, a cell takes some time to rotate into an orientation from which its regular puller-type swimming can resume, by which time it may be in the outer wall layer, some distance away from the wall. A surprising observation is that the distributions of $V_{s},\omega$, and *D_r_* seem to be affected as much for cells approaching the wall as for cells leaving it. One hypothesis is that the steep increase in cell concentration in the outer layer enhances the incidence of cell–cell interactions (“collisions”) through contact between cell bodies and flagella. Such collisions would lead to an increasing number of sharp turns and hence, to an increase in the effective rotational diffusivity. According to standard models of random walks (35), this would cause a corresponding decrease in the effective translational diffusivity *D_T_* (proportional to $V_{s}{}^{2}$), and therefore, if the average concentration *N* remained proportional to $\exp\;(V_{z}z/D_{T})$, where *V_z_* is the vertical component of velocity (proportional to *V_s_*), then *N* would indeed increase.

However, this explanation cannot be the whole story because the maximum concentration is about five times that in the bulk (i.e., approximately $2.5\times 10^{5}\;\text{cells}\;{\text{mL}}^{\text{-}1}$). The region occupied by a single swimming *H. akashiwo* cell can be thought of as a sphere of radius $L_{c}\approx$ 20 µm; the centers of such spheres would be separated by roughly $8L_{c}$, which is too great for hydrodynamic interactions to be very important. Therefore, an additional effect is likely to be involved; a possibility is that *H. akashiwo* cells can respond actively to weak mechanical or chemical disturbances generated by the other cells or the wall (Fig. 3*B*) (36, 37).

In any case, the above arguments ignore the large *θ*-dependence of the swimming behavior. It is possible that a successful continuum model, although presumably valid only for dilute suspensions, would lead to a more satisfactory physical explanation of the findings. Such a model is not yet available because it would require solution of the Fokker–Planck equation for the PDF of swimming orientation, such as that used in refs. 21, 38, and 39, which would need to include the angular dependence of *V_s_*, *ω_w_*, and *D_r_*. This is an important project for the future.

A final observation concerns the behavior near the bottom boundary, *z* = 0, in the vertical plane. It can be seen in Fig. 7*E* that there is an uptick in the cell concentration there. This is not a consequence of sedimentation due to the density difference between the cell body and the ambient fluid because the concentration increase caused by the latter would be independent of *θ*, whereas in our experiments, the high concentration is predominantly associated with swimming orientation approximately parallel to the wall, *θ*  =  0 or ±π (Fig. 7*A*). The phenomenon in our case comes from a subset of the cells that continue to swim downward, on average, even during the light phase [although a substantial portion of them swim downward during the dark phase (40, 41)].

## Materials and Methods

### Cell Culture.

The *H. akashiwo* (GY-H24) cells used in this work (obtained from Shanghai Guangyu Biological Technology Co., Ltd.) originated from the Zhoushan sea area, Zhejiang Province, China. The species was inoculated from an exponential growth-phase culture and incubated for 10 d to reach the concentration of $1.2\times 10^{5}\;\text{cells}\;{\text{mL}}^{\text{-}1}$ using sterile f/2 medium at $25{\;}^{{}^{\circ}}\text{C}$ under a diurnal light cycle (12-h light, 12-h dark) with light intensity of $55.5\;\mu \text{mol}\;{\text{m}}^{-2}\;{\text{s}}^{-1}$. This was the bulk concentration in the culture used in the experiments in the horizontal plane. It was diluted to $5\times 10^{4}\;\text{cells}\;{\text{mL}}^{\text{-}1}$ for the experiments in the vertical plane in order to reduce the potential impact of cell–cell interaction beneath the upper wall due to the enhanced accumulation there.

### Microfluidic Apparatus.

The polydimethylsiloxane (PDMS) channel has a length of 5 cm (*x* direction), width of 1.0 mm (*y* direction), and depth of 0.2 mm (*z* direction; i.e., the negative gravitational direction) (Fig. 1*A*). The PDMS channel was cast using a silicon wafer mold and bonded to a clean cover glass with a thickness of 0.2 mm using plasma-sealed techniques. The interior of the microchannel was treated for 30 min using a 5% solution of bovine serum albumin (BOSF; BSA [fraction V] N0001-25G) in order to prevent cell adhesion to the wall. The culture was injected into the PDMS channel through a conduit using a syringe pump (Pump 11 elite; Harvard Apparatus) and an injection syringe (Hamilton). After the suspensions of *H. akashiwo* were injected into the microchannel, the inlet and outlet of the microchannel were sealed with scotch tape to prevent any possible weak flow driven by evaporation. The whole microfluidic setup was mounted on an optical vibration–isolation platform to minimize the influence of any external perturbation on the cell swimming in the microchannel. The whole experiment was thermally isolated, and no thermal convection occurred; since the channel is tiny, any initial disturbance is also quickly damped. When cell swimming in the vertical plane was investigated, the microfluidic apparatus and imaging system were rotated 90${}^{{}^{\circ}}$ around a horizontal axis so that the focal plane was in the gravitational direction. Despite the increase in cell concentration near the top wall, the chamber was not deep enough for it to trigger gravitational instability and bioconvection; the gyrotactic Rayleigh number, as defined in ref. 42, was O(1−10), much lower than the critical value of $O(10^{3})$.

### Cell Imaging.

The user-customized micro-PIV system (iLA) configured with an inverted fluorescence microscope (IX73; OLYMPUS) was employed to measure cells’ trajectories in the horizontal and vertical planes. Cell imaging was implemented by receiving the fluorescence of 500- to 550-nm wave length induced by a pulsed light-emitting diode (LED) blue light of 450- to 500-nm wave length. The camera and pulsed LED illumination were coordinated using a synchronous controller with time delay less than 1 ns. The imaging window with length of 4 mm and width (or height in the vertical plane) of 1 mm was located in the central region of the whole microchannel; the optical depth of field was 30.8 µm (±15.4 µm from the object plane in focus). To ensure a stable distribution of suspensions, cells’ trajectories were recorded ~20 min after each injection. Cell imaging lasted for 60 s during each sampling based on the controlling parameters of 4× magnification, 10 frames per second, 2,560 × 2,160 pixels, and exposure time of 50 ms. Each pixel represents 1.6 µm, which means that the cell body size (10 µm) is approximately six pixel diameters. Experiments were conducted between 3 and 9 h into the light phase of the 12- to 12-h cycle to rule out the effects of most downward migration of *H. akashiwo*, which takes place during the dark phase as part of the cells’ diurnal migration pattern (40, 41, 43). We observed the sharp turn of *H. akashiwo* near the wall in the horizontal plane using the bright-field mode of an ultrahigh-resolution microscope (DeltaVision OMX V4; GE Healthcare). In this case, the trajectory was recorded based on the controlling parameters of 60× magnification, 200 frames per second, and 1,024 × 1,024 pixels.

### Extraction of Trajectories.

Cells’ trajectories were extracted from the images using the free software ImageJ (https://imagej.nih.gov/ij/index.html) and the plug-in unit TrackMate (https://imagej.net/plugins/trackmate/) (44). Totals of 27,529 and 11,399 trajectories, with mean swimming speed greater than $5\;\mu \text{m}\;{\text{s}}^{-1}$, were extracted to analyze cells’ swimming behaviors and distribution in the horizontal and vertical planes, respectively.

### Data Analysis of Experimental Results.

The trajectories were smoothed five times using 10 points to eliminate their zigzags before computing swimming speed, angular velocity, and rotational diffusivity. The codes used to calculate the number (*N^t^*) and duration (*T^t^*) of sharp turns, as well as the averaged swimming speed ($V_{s}^{t}$) and angular velocity (*ω^t^*) during individual sharp turns, were based on the built-in algorithm (findpeaks) in MATLAB (The Mathworks). The Python program (Python 3.0) together with an open source library (NumPy; https://numpy.org/) was used to compute the variation of *V_s_*, *ω*, *D_r_*, and PDF (*P*) with *y* (or *z*) and orientation ϕ (or *θ*) in the horizontal (or vertical) plane. *V_s_* was computed using a central difference scheme for points in each trajectory, except for the first point using forward differences and the last point using backward differences. To obtain the rotational diffusivity in the horizontal plane, we assume each cell obeys the stochastic differential equations (Eq. **2**). Thus, for a time step $\Delta t,\;\Delta \phi \approx \omega (y,\phi )\Delta t+\Delta \phi^{\text{B}}$, where $\Delta \phi^{\text{B}}$ is the diffusion part (modeled as rotational Brownian motion) subject to $\Delta \phi^{\text{B}}\sim \hat{N}(0,2D_{r}(y,\phi )\Delta t)$, where $\hat{N}$ is the normal distribution. Therefore, we can use the SD, *σ*, of Δϕ at each *y* and ϕ to calculate $D_{r}(y,\phi )\approx \sigma^{2}/(2\Delta t)$. We have checked the convergence of this calculation for rotational diffusivity. Convergence seems to be approached as Δt is reduced to 0.1 s; we cannot take it lower because that is the time between experimental frames (*SI Appendix*, Fig. S11). From the large number of trajectories obtained in our experiments, we calculate Δϕ and perform a two-dimensional histogram statistic over *y* and ϕ. For each bin, we calculate ω(y,ϕ) and $D_{r}(y,\phi )$ from the first- and second-order moments of Δϕ, respectively. The resulting data were used to generate a map of *V_s_*, *ω*, *D_r_*, and *P* with orientation and position (51  ×  80 rectangles). The same procedure was performed to obtain the angular velocity and rotational diffusivity of cells swimming in the vertical plane.

### Mathematical Model.

The individual model in the vertical plane is given by

[3] $$\{\begin{array}{l} \frac{\text{d}z}{\text{d}t}=-V_{s}(z,\theta )\sin\;\theta ,\\ \text{d}\theta =\underset{\omega (z,\theta )}{\underset{︸}{\left[\omega_{g}(z,\theta )+\omega_{w}(z,\theta )\right]}}\text{d}t+\sqrt{2D_{r}(z,\theta )}\text{d}W, \end{array}$$

where *ω_g_* and *ω_w_* are the angular velocities induced by gyrotaxis and cell-wall interaction, respectively. The individual models (Indi. models I to V) with different terms omitted are used to explore the physics behind the observations: Indi. model I: *V_s_* (cell-wall interaction), *ω* (*ω_w_*, cell-wall interaction), and *D_r_* (constant); Indi. model II: *V_s_* (cell-wall interaction), *ω* (*ω_w_*, cell-wall interaction), and *D_r_* (cell-wall interaction); Indi. model III: *V_s_* (constant), *ω* (*ω_g_*, gyrotaxis), and *D_r_* (constant); Indi. model IV: *V_s_* (cell-wall interaction), *ω* ($\omega_{g}\;+\;\omega_{w}$, gyrotaxis plus cell-wall interaction), and *D_r_* (constant); Indi. model V: *V_s_* (cell-wall interaction), *ω* ($\omega_{g}\;+\;\omega_{w}$, gyrotaxis plus cell-wall interaction), and *D_r_* (cell-wall interaction).

### Numerical Algorithm.

The numerical algorithm and time step are the same for modeling of cell migration in the horizontal and vertical planes. We apply a forward Euler scheme with time step $\Delta \tau =2\times 10^{-4}$ for time marching, where $\tau =t\;V_{sb}/H$. The instantaneous *V_s_*, *ω*, and *D_r_* during each time step are interpolated from the experimental results using a linear interpolation scheme, except those that are specified as constant or given by an expression (angular velocity due to gyrotaxis) to examine their contribution. A reflection boundary condition is employed to determine the outgoing angle and velocity from the walls $y/L_{c}$ (or $z/L_{c}$) = 0 and 50, and a periodic boundary condition is used for *P* at ϕ (or *θ*) =±π. The numerical algorithm was implemented using the Python program (Python 3.0) together with the NumPy library (https://numpy.org/). Cells are initially located at $y/L_{c}$ (or $z/L_{c}$) = 25 with ϕ (or *θ*) uniformly distributed in the interval (−π,π). 10^5^ trajectories were simulated until *τ*  =  10 during each simulation.

## Supplementary Material

Supplementary File

Supplementary File

## Data Availability

Simulation code has been deposited in Zenodo (https://zenodo.org/record/7071215) (45). All other data are included in the article and/or supporting information.
