# Supplementary material for: Sharp turns and gyrotaxis modulate surface accumulation of microorganisms
Source: Proc Natl Acad Sci U S A. 2022 Oct 11;119(42):e2206738119. doi: 10.1073/pnas.2206738119 (PMC9586295; doi:10.1073/pnas.2206738119)
Supplement: Supplementary File [file pnas.2206738119.sapp.pdf]

PNAS
www.pnas.org

Supplementary Information for

Sharp turns and gyrotaxis modulate surface accumulation of microorganisms

Li Zeng, Weiquan Jiang and Timothy J. Pedley

T.J. Pedley.
E-mail: tjp3@cam.ac.uk

This PDF file includes:

Figs. S1 to S11 (not allowed for Brief Reports)
Legend for Movie S1

Other supplementary materials for this manuscript include the following:

Movie S1

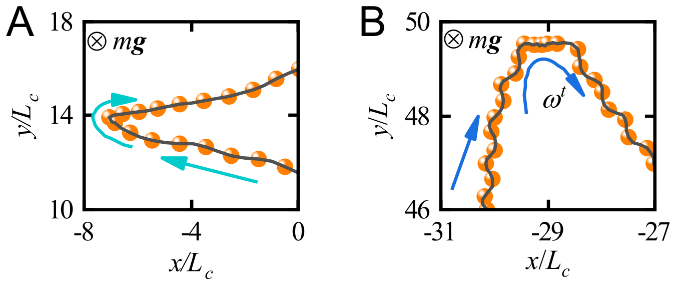

Fig. S1. Expanded view of sharp turns in the horizontal plane (x-y). (*A*) The sharp turn in the bulk marked by the black square in Fig. 1*B*. (*B*) The sharp turn near the wall marked by the red square in Fig. 1*B*. The time intervals of two consecutive points are 0.2 s and 0.3 s in (*A*) and (*B*), respectively. The cyan and blue straight arrows in (*A*) and (*B*) represent the travel direction of cells, while the curly arrows represent the rotational direction during the sharp turns. After the first sharp turn near the wall, a cell tends to slip on the wall, while, in the bulk, it moves freely in an arbitrary direction.

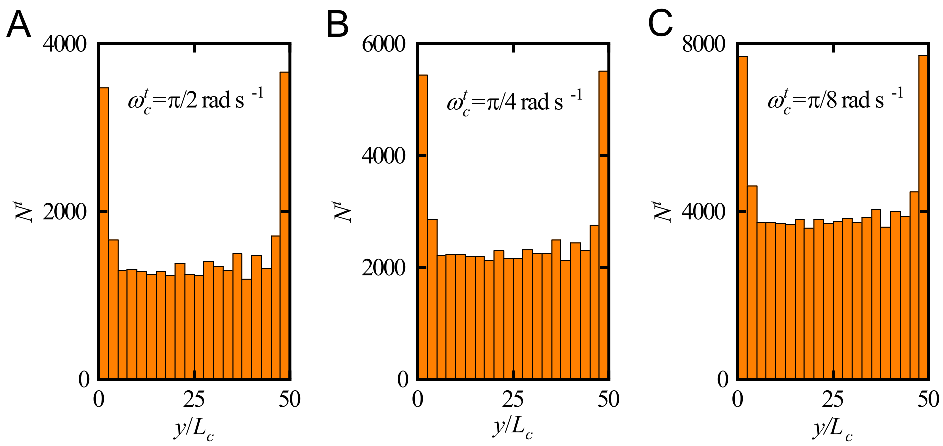

Fig. S2. The number of sharp turn events, N^t, occurring at different locations across the channel, y/L_c. A sharp turn is supposed to occur when the magnitude of angular velocity exceeds a critical value ω_c^t, which is much greater than the mean value of the magnitude of angular velocity $(0.04\ \mathrm{rad\ s^{-1}})$ in bulk. (*A*), (*B*) and (*C*) present the the variation of N^t with y/L_c for $\omega_c^t = \pi/2\ \mathrm{rad\ s^{-1}}$, $\pi/4\ \mathrm{rad\ s^{-1}}$, and $\pi/8\ \mathrm{rad\ s^{-1}}$, respectively. It is shown that the phenomenon that more sharp turns occur near the wall is qualitatively the same for three ω_c^t.

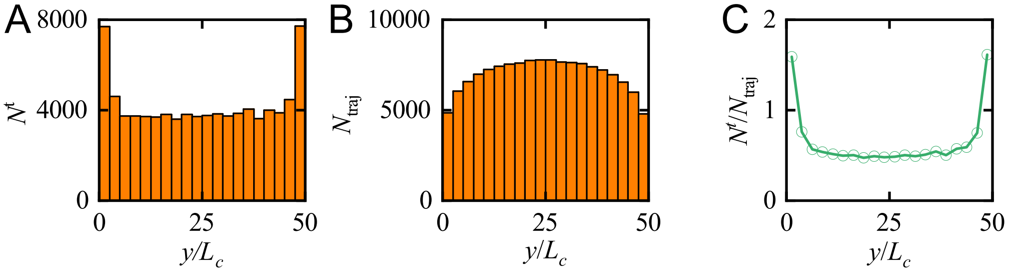

Fig. S3. The relationship between the number of sharp turn events, N^t, and the number of trajectories, N_{traj}, occurring at different locations across the channel, y/L_c. (*A*), (*B*) and (*C*) present the the variation of N^t, N_{traj}, and N_t/N_{traj} with y/L_c, respectively. It is shown that the increase of sharp turn events is caused by the enhanced frequency of sharp turns due to the existence of the wall, rather than the increase of cell numbers.

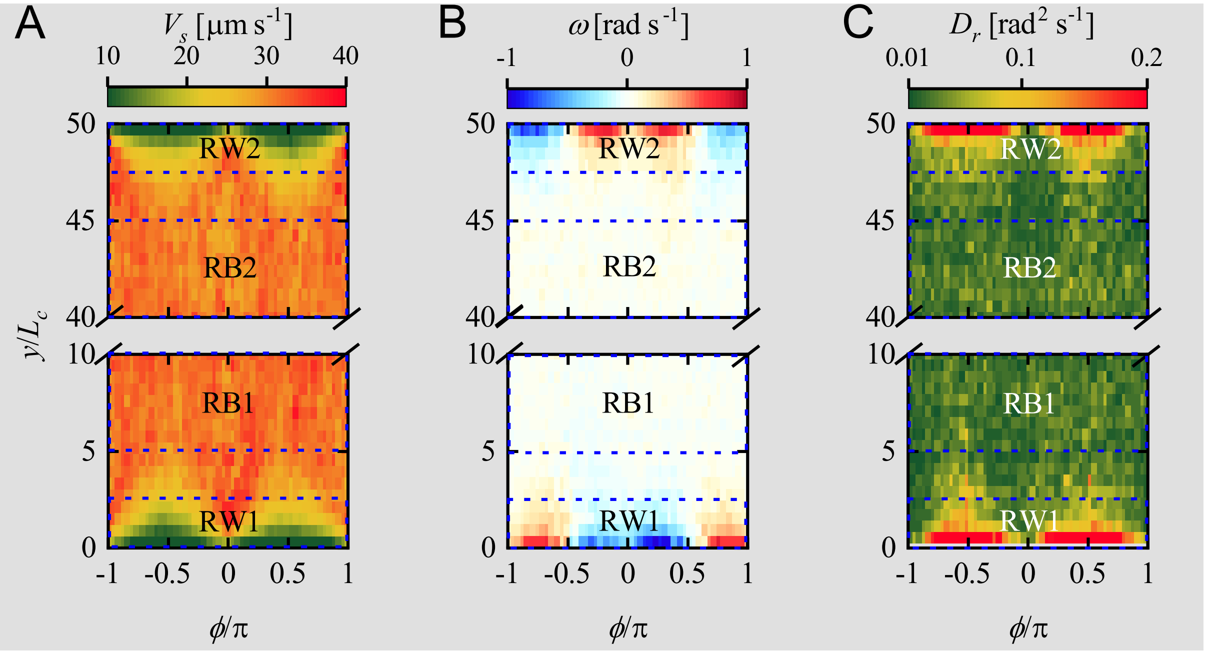

Fig. S4. Swimming behaviour of *H. akashiwo* in the horizontal plane. The variation of (*A*) the ensemble averaged swimming speed V_s, (*B*) angular velocity ω, and (*C*) rotational diffusivity D_r, with the lateral position y/L_c and swimming direction ϕ/π. RW2 and RW1 denote the regions next to the wall, $0 \leq y/L_c \leq 2.5$ and $47.5 \leq y/L_c \leq 50$, respectively. RB1 and RB2 denote the regions almost unaffected by the wall, $5 \leq y/L_c \leq 10$ and $40 \leq y/L_c \leq 45$, respectively.

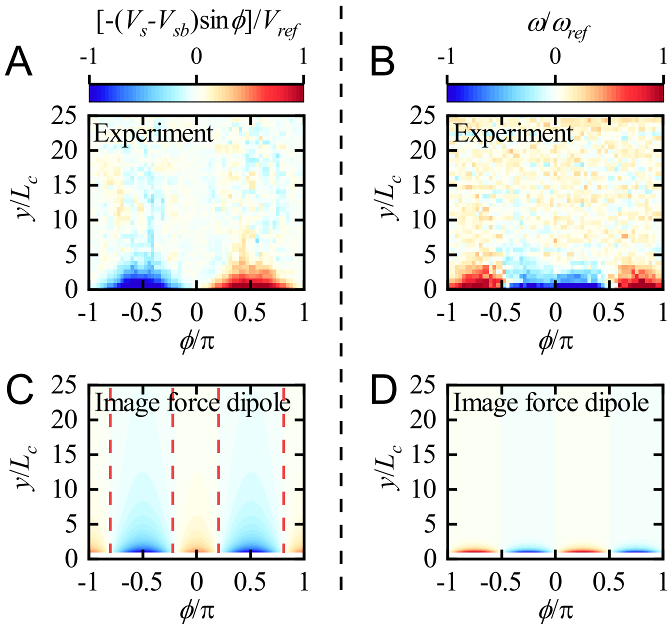

Fig. S5. Comparison of induced swimming behaviours by the wall of $y/L_c = 0$ given by the tilted image force-dipole theory (*C* and *D*) and experimental observation (*A* and *B*). (*A* and *C*) The dimensionless induced velocity component perpendicular to the wall, $-(V_s - V_{sb})\sin\phi/V_{\mathrm{ref}}$, where V_{ref} is the maximum induced velocity in the region of $1 \le y/L_c \le 25$. The red dash lines denote the orientations of $\phi = -\pi/2 - \arccos(1/\sqrt{3})$, $-\pi/2 + \arccos(1/\sqrt{3})$, $\pi/2 - \arccos(1/\sqrt{3})$, and $\pi/2 + \arccos(1/\sqrt{3})$, at which the conversion of attraction and repulsion occurs, i.e. the sign of induced velocity changes. (*B* and *D*) The dimensionless induced angular velocity, $\omega/\omega_{\mathrm{ref}}$, where ω_{ref} is the maximum angular velocity in the region of $1 \le y/L_c \le 25$. For an incoming swimmer ($0 \le \phi/\pi \le 1$), the angular velocity given by the tilted image force-dipole theory is qualitatively inconsistent with the experimental result.

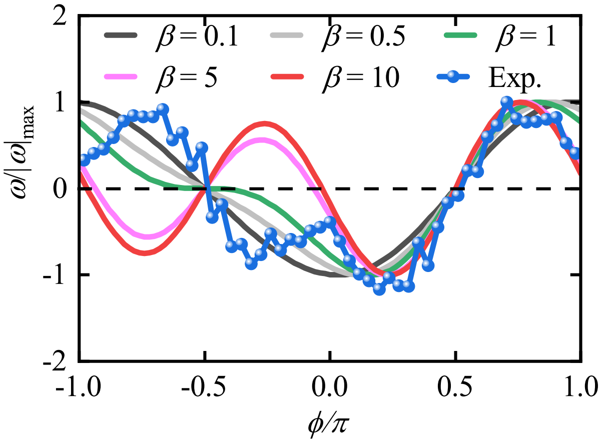

Fig. S6. Comparison of the normalized ensemble-averaged angular velocity of *Heterosigma akashiwo* very close to the wall ($y/L_c \leq 0.625$) given by the lubrication theory and experimental (Exp.) observation. $|\omega_{\max}|$ is the maximum value of the ensemble-averaged angular velocity ω, and β reflects the strength of a puller. The variation of $\omega/|\omega_{\max}|$ with the orientation, ϕ, cannot be well described by the lubrication results, especially the angle corresponding to the maximum angular velocity.

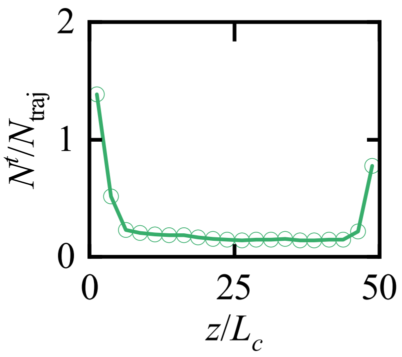

Fig. S7. The variation of N^t/N_{traj} with vertical locations z/L_c, where L_c $(20\,\mu\mathrm{m})$ is the characteristic length of a cell approximately equal to half the cell body length plus the length of one flagellum, and N^t and N_{traj} represent the number of sharp turn events and trajectories in each space of L_c, respectively. It is shown that the probability of sharp turns is caused by the wall interaction, not by the increased cell concentration.

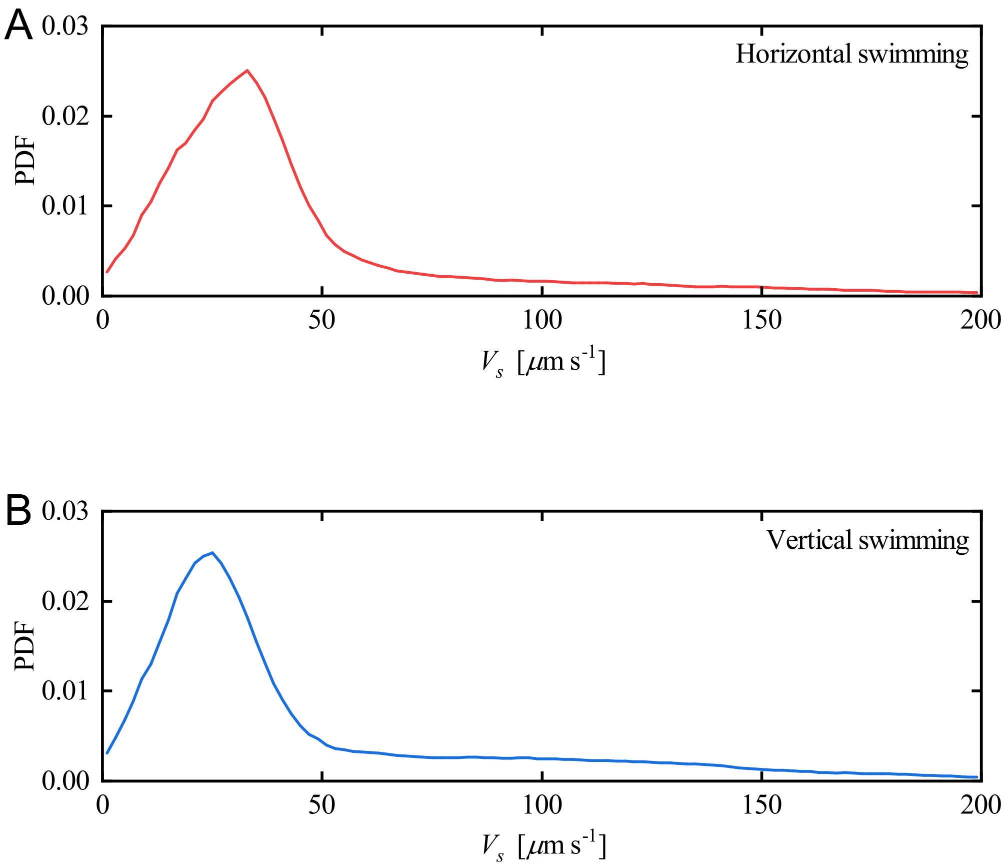

Fig. S8. Comparison of swimming speed in the horizontal and vertical planes. (*A*) Variation of probability density function (PDF) of V_s in the horizontal plane. (*B*) Variation of probability density function (PDF) of V_s in the vertical plane. The measured swimming speed is comparable for all cells in the whole width and height.

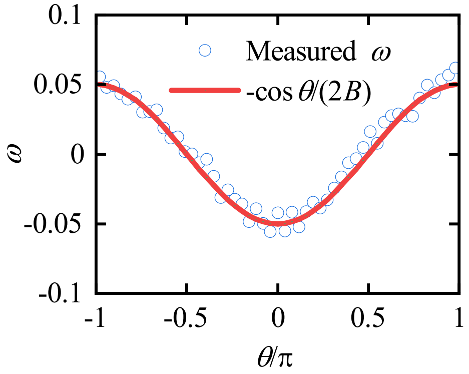

Fig. S9. The variation of angular velocity ω of cells in the bulk with their orientation θ for the vertical plane. The blue circles denote the measured angular velocity in the bulk region. The red line denotes the fitted curve using the formula $-\cos\theta/(2B)$, which is the specific expression of the angular velocity due to gyrotaxis $\boldsymbol{\omega}_g = [\boldsymbol{k} - (\boldsymbol{k}\cdot\boldsymbol{p})\boldsymbol{p}]/(2B)$, where $\boldsymbol{p}$ is the swimming direction, $\boldsymbol{k}$ is the unit vector directed vertically upwards, and B stands for the timescale for cell reorientation by the gravitational torque against the viscous resistance ($B \approx 10$ s here).

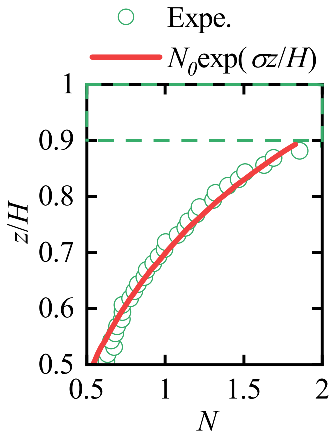

Fig. S10. Variation of cell concentration, N, in the bulk with z in the vertical plane. The green rectangle represents the region of cell distribution affected by cell-wall interaction. The green circles denote the measured cell concentration, and the red line denotes the fitted concentration using the exponential formula $N = N_0 \exp(\sigma z/H)$ for constant N_0 and $\sigma = V_z H/D_T$, where D_T is the effective translational diffusivity and V_z is the vertical cell velocity in the bulk, both assumed to be constant. This formula agrees well with the experimental curve if σ, an effective Péclet number, is equal to 3.112.

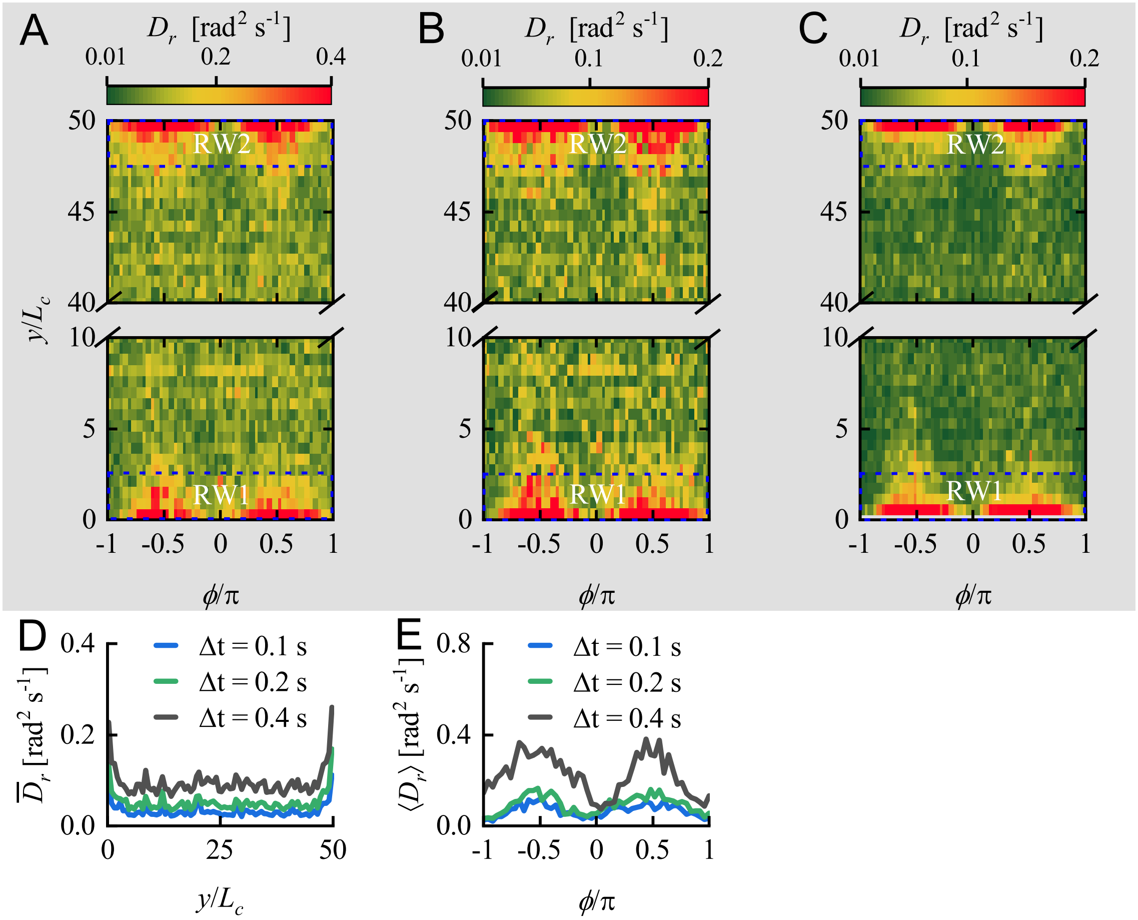

Fig. S11. Distribution of rotational diffusivity, D_r. The variation of D_r with the lateral position y/L_c and swimming direction ϕ/π for various sampling intervals (*A*) $\Delta t = 0.1$ s, (*B*)$\Delta t = 0.2$ s, and (*C*) $\Delta t = 0.4$ s. RW1 and RW2 denote the regions next to the wall, $0 \leq y/L_c \leq 2.5$ and $47.5 \leq y/L_c \leq 50$, respectively. (*D*) Variation of the orientation-averaged rotational diffusivity, $\bar{D}_r$, with y/L_c for various sampling intervals $\Delta t = 0.1$ s (blue line), $\Delta t = 0.2$ s (green line), and $\Delta t = 0.4$ s (gray line). (*E*) Variation of the width-averaged rotational diffusivity , $\langle D_r \rangle$, for near-wall regions RW1 and RW2 with ϕ/π for various sampling interval $\Delta t = 0.1$ s (blue line), $\Delta t = 0.2$ s (green line), and $\Delta t = 0.4$ s (gray line).

Movie S1. A sharp turn of *Heterosigma akashiwo* in the region close to a wall. The wall greatly affects the motility of both forward and trailing flagella. The video was captured by ultrahigh resolution microscope (DeltaVision OMX V4, GE Healthcare, US), based on the controlling parameters of ×60 magnification, 200 frames per second, and 1024×1024 pixels. The video is played back 32 times slower than real time.
